# Does chemotherapy improve survival in patients with nodal positive luminal A breast cancer? A retrospective Multicenter Study

**DOI:** 10.1371/journal.pone.0218434

**Published:** 2019-07-08

**Authors:** Daniel Herr, Manfred Wischnewsky, Ralf Joukhadar, Olivia Chow, Wolfgang Janni, Elena Leinert, Visnja Fink, Tanja Stüber, Carolin Curtaz, Rolf Kreienberg, Maria Blettner, Daniel Wollschläger, Achim Wöckel

**Affiliations:** 1 Department of Obstetrics and Gynaecology, Würzburg University Medical Centre, Würzburg, Germany; 2 Faculty of Mathematics and Computer Science, University of Bremen, Bremen, Germany; 3 Department of Obstetrics and Gynaecology, Ulm University Medical Centre, Ulm, Germany; 4 Institute of Medical Biostatistics, Epidemiology and Informatics, University Medical Center, Mainz, Germany; Institut national de la recherche scientifique, CANADA

## Abstract

**Background:**

In this study based on the BRENDA data, we investigated the impact of endocrine ± chemotherapy for luminal A, nodal positive breast cancer on recurrence free (RFS) and overall survival (OS). In addition, we analysed if tumor size of luminal A breast cancer influences survival in patients with the same number of positive lymph nodes.

**Methods:**

In this retrospective multi-centre cohort study data of 1376 nodal-positive patients with primary diagnosis of luminal A breast cancer during 2001–2008 were analysed. The results were stratified by therapy and adjusted by age, tumor size and number of affected lymph nodes.

**Results:**

In our study population, patients had a good to excellent prognosis (5-year RFS: 91% and tumorspecific 5-year OS 96.5%). There was no significant difference in RFS stratified by patients with only endocrine therapy and with endocrine plus chemo-therapy. Patients with 1–3 affected lymph nodes had no significant differences in OS treated only with endocrine therapy or with endocrine plus chemotherapy, independent of tumor size. Patients with large tumors and more than 3 affected lymph nodes had a significant worse survival as compared to the small tumors. However, despite the worse prognosis of those, adjuvant chemotherapy failed in order to improve RFS.

**Conclusions:**

According to our data, nodal positive patients with luminal A breast cancer have, if any, a limited benefit of adjuvant chemotherapy. Tumor size and nodal status seem to be of prognostic value in terms of survival, however both tumor size as well as nodal status were not predictive for a benefit of adjuvant chemotherapy.

## Introduction

In this day an age, women with the primary diagnosis of breast cancer all inall have a favourable prognosis with good survival rates. However, despite huge efforts in breast cancer research during the last 20 years, still a significant percentage of patients will suffer metastatic disease. Unfortunately, down to the present date metastatic breast cancer is still incurable. Apparently, from a theoretical point of view, at the time of first diagnosis, there are three different groups of patients: First of all those patients, which do not benefit from any adjuvant therapy such as endocrine- chemo- or immunotherapy. This small group of patients will develop metastasis and reach an incurable stage of cancer irrespective of the administered adjuvant therapy. Secondly, those patients who will survive anyway, even without any adjuvant therapy. The last and presumably biggest group of patients is able to benefit from adjuvant therapy and their survival rates are susceptible by adjuvant treatment. However, in almost all cases it is not possible to predict at the time of primary diagnosis, to which of those three groups a breast cancer patient will belong to. Due to this fact, even within a group of patients with good prognosis, up do date all patients receive the same therapy.

In order to evaluate the individual risk in breast cancer patients, during the last 20 years, conventional prognostic factors such as nodal status or tumor size have been investigated extensively. However, multiple studies revealed that breast cancer is a heterogenous disease with different intrinsic subtypes and gene expression profiles [1–4]. The St. Gallen panel of 2011 defined five different subtypes of breast cancer. The five subtypes were: Luminal A, luminal B (human epidermal growth factor receptor 2 (HER2) negative), luminal B (HER2 positive), triple negative and HER2-overexpressing. The expert panel as well as the german S3-guideline provided systemic treatment recommendations for the subtypes including endocrine therapy alone for luminal A, endocrine and chemotherapy for luminal B (HER2 negative); chemotherapy and anti-HER2-therapy as well as endocrine therapy for luminal B (HER2 positive); chemotherapy and anti-HER2-therapy for HER2 overexpressing (non luminal); and chemotherapy for triple negative cancers [5, 6].

Luminal A subtype is the most common molecular subtype with an explicit gene expression [7, 8]. Luminal A breast cancer is hormone-receptor positive (HR+), HER2 negative (HER2-) and has a low expression of the cell proliferation marker Ki-67 [5, 9, 10]. Since from a clinical point of view, those patients with luminal A breast cancer have an excellent prognosis in general, adjuvant endocrine therapy is well established [10]. However, the impact of adjuvant chemotherapy is questionable, since the benefit on survival seems to be small. Therefore, in case of lacking further risk factors such as advanced tumor size or positive lymph nodes, the renunciation of adjuvant chemotherapy is clinical standard. In case of higher risk concerning those conventional criteria, there is lack of data, since the majority of clinical chemotherapy trials during the last decades did not distinguish the intrinsic subtypes of cancer. For this reason, there is a considerable demand to clarify the question, of adequate adjuvant therapy in patients with nodal positive, luminal A cancer. In this study based on the BRENDA data, we investigated the impact of endocrine ± chemotherapy for luminal A, nodal positive breast cancer on recurrence free (RFS) and overall survival (OS). In addition, we analysed if tumor size of luminal A breast cancer influences survival in patients with the same number of positive lymph nodes.

## Patients and methods

In this retrospective multi-centre cohort study of the BRENDA (= breast cancer care under evidence-based guidelines) study group, we extracted data from 1376 nodal-positive patients with luminal A breast cancer patients at the Department of Gynecology and Obstetrics at the University of Ulm and 16 partner hospitals (all certified breast cancer centres) in Baden-Wuerttemberg (Germany) for the period 2001–2008. The exact conditions and inclusion criteria of BRENDA have been described previously [11, 12]. Patients receiving chemotherapy have been administered to antracyclins and/or taxans. Endocrine treatment included tamoxifen and/or aromatase inhibitors. the follow-up, data on first recurrences, secondary tumors, survival status and date as well as the cause of death were collected. As measures of comorbidity, the American Society of Anesthesiologists Physical Status (ASA) and the New York Heart Association cardiac score (NYHA) were collected for all patients at the time of surgery. Written and informed consent was obtained from all patients included in this clinical study.

Surrogate definition of luminal A: Because information on Ki-67 was not available, we used grade as a surrogate parameter to include the cell proliferation, as described before e.g. by others [1, 13–15]. With grade instead of Ki-67 luminal A is defined by HR+, HER2− and tumor grade 1 or 2 (118 tumors with grade 1 and 1258 tumors with grade 2). Staining for ER/PR/Her2 was performed-on pre-op specimens.

### Menopausal status

Patients confirming the following conditions have been considered as postmenopausal: women older than 60 years, women with a history of bilateral ovariectomy, and women being amenorrheic for at least one year prior to the diagnosis of breast cancer. All patients having regular menses without using oral contraceptives or HRT are classified as premenopausal.

### Statistical analysis

All categorical data were described using numbers and percentages. Comparisons of categorical variables between groups were made using χ2 tests. Quantitative data were presented using median and range or mean and standard deviations. The primary endpoint was relapse-free survival (RFS), which was assessed by a standard survival analysis using the non-parametric Kaplan-Meier approach. Relapse–free survival is defined as any disease recurrence (local, regional, or distant), but death is censored (not included). If a patient was lost to follow-up, data were censored at the date of the last known contact. When no information was available, the status was coded as missing data. Survival distributions and median survival times were estimated using the Kaplan–Meier product-limit method. The log rank-test was used to provide a formal statistical assessment of the differences between treatment arms. The 5 and 10-year survival rates with 95% confidence interval (95% CI) were computed using Kaplan-Meier product-limit survival probabilities at the specified time points. The Cox proportional hazards model adjusted for age, tumor size etc. was used to estimate the hazard ratio (HR) and 95% confidence intervals. A test of the PH assumption was performed for each covariate and globally using a formal significance test based on the unscaled and scaled Schoenfeld residuals. In addition, we used propensity score methods. Propensity score methods try to approximate a (sometimes fully blocked) randomized experiment. The goal was to reduce imbalance in the empirical distribution of the pre-treatment confounders like age, number of affected lymph nodes or tumor size between the only endocrine and the endocrine plus chemotherapy groups to generate approximately unbiased “treatment” effect estimates in our observational study. We estimated adjusted survival curves and log-rank test based on inverse probability weighting (IPW). The weights were calculated by using logistic regression. The adjusted survival curves were computed by weighting the individual contributions by the inverse of the probability to be in the group endocrine therapy or endocrine plus chemotherapy. The usual log-rank test was adapted to the corresponding adjusted survival. All statistical tests were two-sided. The level of statistical significance was set at 0.05. Statistical analyses were carried out with R 3.5, SPSS 25 and NCSS 10.

## Results

### Study population

This analysis includes a total number of 1376 nodal-positive, luminal A breast cancer patients. 493 (35,8%) received only anti-hormonal therapy and 883 patients (64,2%) anti-hormonal- and chemotherapy. Mean age of all patients was 62,7 years (range 28–98), 75 years (range 38–98) in the group of patients only treated with anti-hormonal therapy, and 57 years (range 28–82) in the anti-hormonal- and chemotherapy-treated group (p>0.001). 610 (44,3%) patients had T1 tumors and 766 (55,7%) T>2cm. There were no significant differences concerning the treatment arms: 205 only anti-hormonally treated patients (41,6%) with T1 tumors vs. 288 (58,4%) T2 tumors, and 405 (45%) anti-hormonal and chemotherapy treated patients with T1 tumors vs. 381 (39%) T2 tumors in this group. In the total study population 1047 (76,1%) women were postmenopausal. However, in the only anti-hormonally treated group 464 women (94,1%) and in the anti-hormonal- and chemotherapy-treated population 583 (66%) has been considered as postmenopausal (p>0.001). The nodal status was distributed as follows: In the total study population 907 women (65,9%) had 1–3 positive lymph nodes, and 469 women (34,1%) with more than three positive lymph nodes. The anti-hormonally treated group of patients was consisting of 359 women (72,8%) with 1–3 positive lymph nodes, and 134 women (27,2%) with more than 3 positive lymph nodes. Expectedly, the patients in the anti-hormonal- and chemotherapy-treated population had a smaller proportion of 1–3 positive (548 women, 62,1%) as compared to more than 3 positive lymph nodes (335 women, 37,9%) (p<0.001). The assignment of risk in matters of the Nottingham Prognostic Index is illustrated in Table 1.

**Table 1:** Basic characteristics of the study population.

Luminal A, nodal positive patients					p-value
		Total	H	H+C	
		1376	493 (35.8)	883 (64.2)	
**Age at primary diagnosis**		mean: 62.7 (SD 13.1) (median:63)	mean: 72.8 (SD 11.1) (median:75)	mean: 57.1 (SD 10.4)	<0.001
				(median: 57)	
		Range: 28–98	Range: 38–98	Range:28–82	
**T-categories**	**T1**	610 (44.3)	205 (41.6)	405 (45.)	0.125
	**T > 2cm**	766 (55.7)	288 (58.4)	1381 (39.0)	
**Menopausal status**	**pre**	292 (21.2)	26 (5.3)	266 (30.1)	< 0.001
	**peri**	37 (2.7)	3 (0.6)	34 (3.9)	
	**post**	1047 (76.1)	464 (94.1)	583 (66.0)	
**Nodal staus**	**1–3 affected lymph nodes**	907 (65.9)	359 (72.8)	548 (62.1)	< 0.001
	**> 3 affected lymph nodes**	469 (34.1)	134 (27.2)	335 (37.)	
**Nottingham Prognostic Index**	**low risk**	62 (4.5)	30 (6.1)	32 (3.6)	0.043
	**intermediate risk**	1003 (73.0)	364 (73.8)	639 (72.5)	
	**high risk**	309 (22.5)	99 (20.1)	210 (23.8)	

H = endocrine therapy; C = chemotherapy. It is evident, that the mean age in the group of patients solely treated with endocrine therapy is significantly higher as compared to those treated with endocrine- and chemotherapy.

### Outcome of the entire study population (endocrine therapy and chemotherapy/ endocrine therapy)

Recurrence free survival (RFS) of all 1376 patients with nodal positive luminal A tumors stratified by therapy and adjusted by age, tumor size and number of affected lymph nodes taking into account interactions between covariates revealed no significant differences between the two groups (p = 0,167; HR = 1.32, 95% CI 0.89–1.96): 5-year RFS was 91% in the only hormonally treated group and 92,5% in patients with chemo- and endocrine therapy.

**Fig 1:**
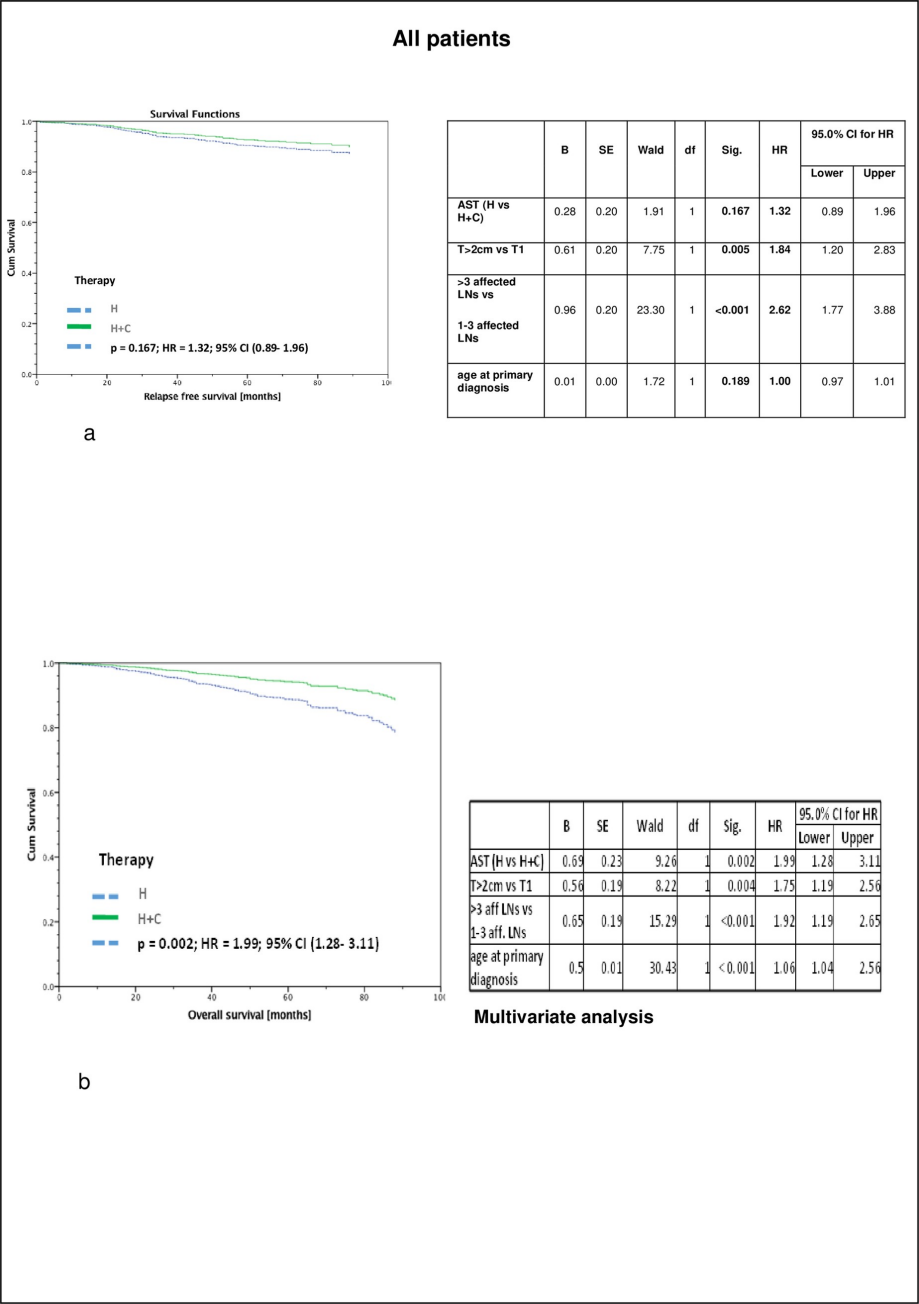
All patients. a) Recurrence free survival (RFS) of Luminal A, nodal positive patients (irrespective of the number of positive lymph nodes), stratified by therapy and adjusted by age, tumor size and number of affected lymph nodes taking into account interactions between covariates, revealing no significant differences between the two groups (5-year RFS: H 91%; H + C 92,5%) n = 1376. b) Overall survival (OAS) of Luminal A, nodal positive patients (irrespective of the number of positive lymph nodes), stratified by therapy and adjusted by age, tumor size and number of affected lymph nodes taking into account interactions between covariates revealing a significant different survival between the two groups (5-year OS: H 89%, H + C 94%), n = 1376.

However, with regard to overall survival (OS) of the same population, significant differences between both treatment groups have been observed (p = 0,002; HR = 1.99, 95% CI 1.28–13.11). Despite the positive nodal status of those patients, 5-year OS was 89% (endocrine therapy) vs. 94% (chemo- and endocrine therapy) (p = 0,002) (Fig 1).

### Survival of patients with 1–3 positive lymph nodes

Regarding the subgroup of 907 patients with 1–3 positive axillary lymph nodes (all therapies), it becomes apparent, that neither RFS nor OS was significantly different as a function of tumor size (T1 vs. T>2cm): 5-year RFS in T1-tumors was 95,2% vs. 92,8% in tumors T> 2cm and 5-year OS was 95,6% in T1-tumors vs. 94,8% in T>2cm.

**Fig 2:**
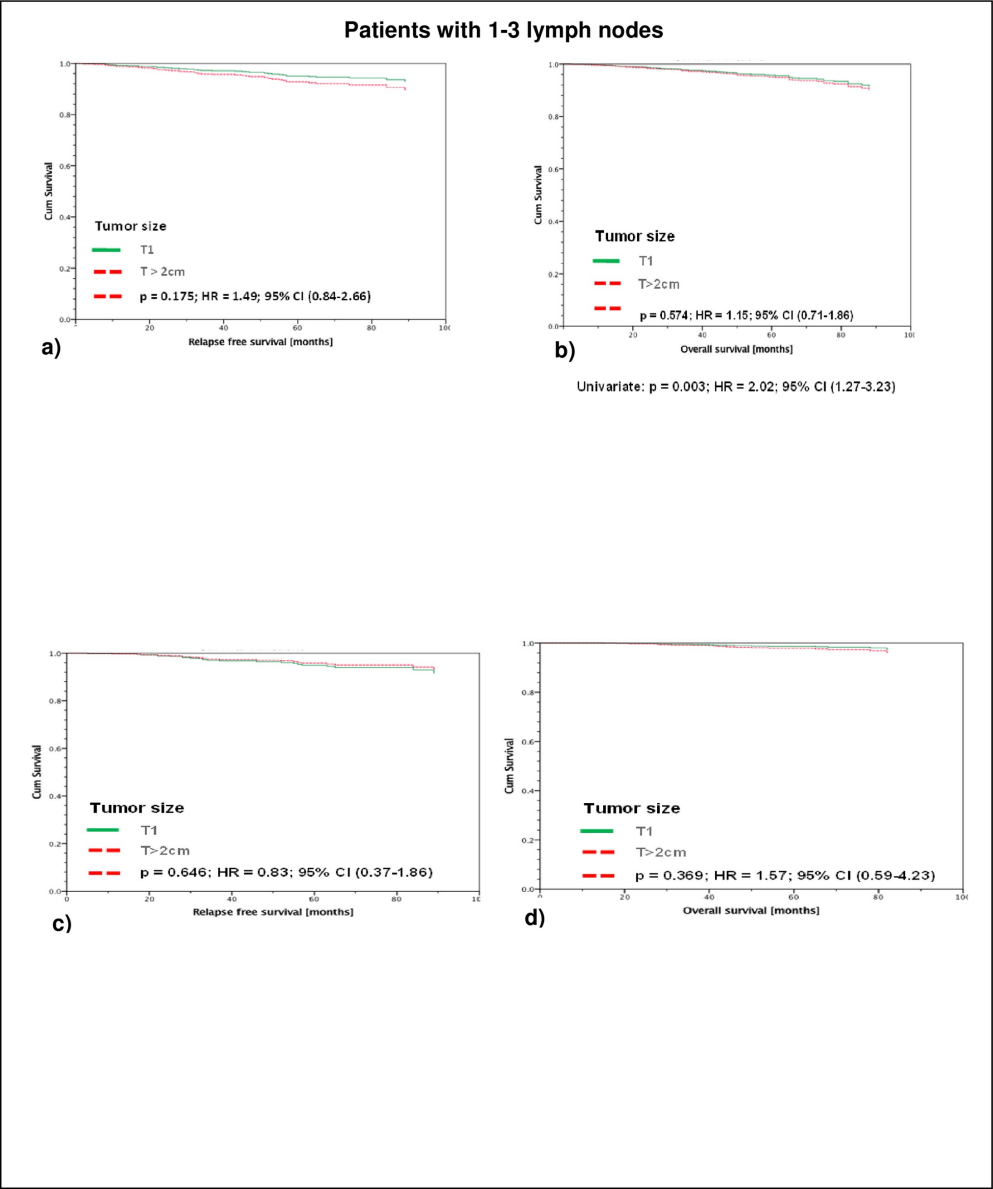
Patients with 1–3 lymph nodes. Legend a -b: RFS (a) and OAS (b) of all Luminal A patients with ≤ 3 affected lymph nodes, stratified by tumor size and adjusted by therapy and age. There is no significant difference in RFS or OAS for the T1 tumors as compared to the T>2 cm (5-year RFS: T1 95,2%; T> 2cm 92,8%; 5-year OS: T1 95,6%; T>2cm 94,8%), n = 907. Legend c-d: RFS (c) and OAS (d) of only those of the total of 907 Luminal A patients with ≤ 3 affected lymph nodes which had received adjuvant endocrine- and chemotherapy, stratified by tumor size and adjusted by therapy and age. Again, there is no significant difference in RFS or OAS for the T1 tumors as compared to the T>2 cm (5-year RFS: T1 94,9%; T>2cm 95,6%; 5-year OAS: T1 98,6%; T>2cm 97,8%), n = 548.

Beyond that, the group of 548 patients having received both, adjuvant chemotherapy and adjuvant endocrine treatment, again no difference in RFS and OS between T1 and T>2cm tumors could be shown. 5-year RFS was 94,9% vs. 95,6% (T1 and T>2cm) and 5-year OS was 98,6% vs. 97,8% (T1 vs. T>2cm) (Fig 2A and 2B).

### Survival of patients with > 3 positive lymph nodes

**Fig 3:**
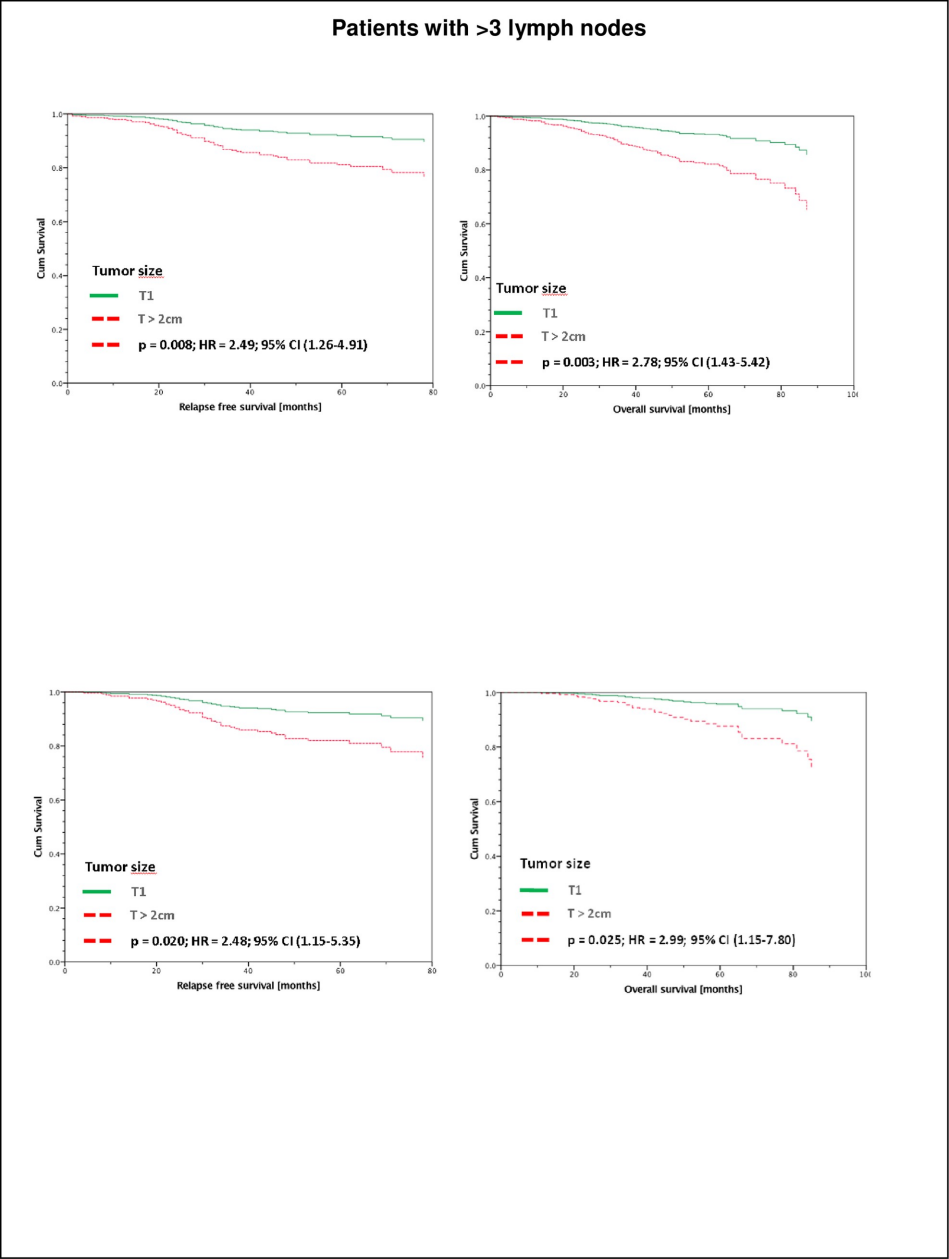
Patients with >3 lymph nodes. RFS (a) and OAS (b) of all Luminal A patients with > 3 affected lymph nodes stratified by tumor size and adjusted by therapy and age. In this group of > 3 affected lymph lodes, patients with tumors > 2cm have a significant worse survival (5-year RFS: T1 92,0%; T>2cm 81,2%; 5-year OAS T1 93,2%; T>2cm 82,1%), n = 469. RFS (c) and OAS (d) of only those of the total of 469 Luminal A patients with > 3 affected lymph nodes which had received adjuvant endocrine- and chemotherapy, stratified by tumor size and adjusted by therapy and age. In this group of > 3 affected lymph lodes, patients with tumors > 2cm have a significant worse survival (5-year RFS: T1 92,5%; T>2cm 82,0%; 5-year OAS T1 96,0%; T>2cm 87,8%), n = 335.

Interestingly, RFS as well as OS of those 469 patients with more than 3 positive axillary lymph nodes and tumors >2cm (all therapies) was significantly worse as compared to T1 tumors (5-year RFS: T1 92,0% and T>2cm 81,2%; p>0,003; 5-year OS in T1 93,2% and T>2cm 82,1%; p>0,008 (Fig 3A and 3B).

Furthermore, the 335 patients of the mentioned 469 patients with more than 3 positive lymph nodes having received both, adjuvant chemotherapy and adjuvant endocrine treatment also had a significantly worse RFS and OS in tumors T>2cm vs. T1 (5-year RFS: T1 92,5% and T>2cm; p>0,025; 82,0%; 5-year OAS in T1 96,0% and T>2cm 87,8%; p>0,02) (Fig 3C and 3D)

### Impact of adjuvant chemotherapy in patients with tumors T>2cm and > 3 positive lymph nodes

**Fig 4:**
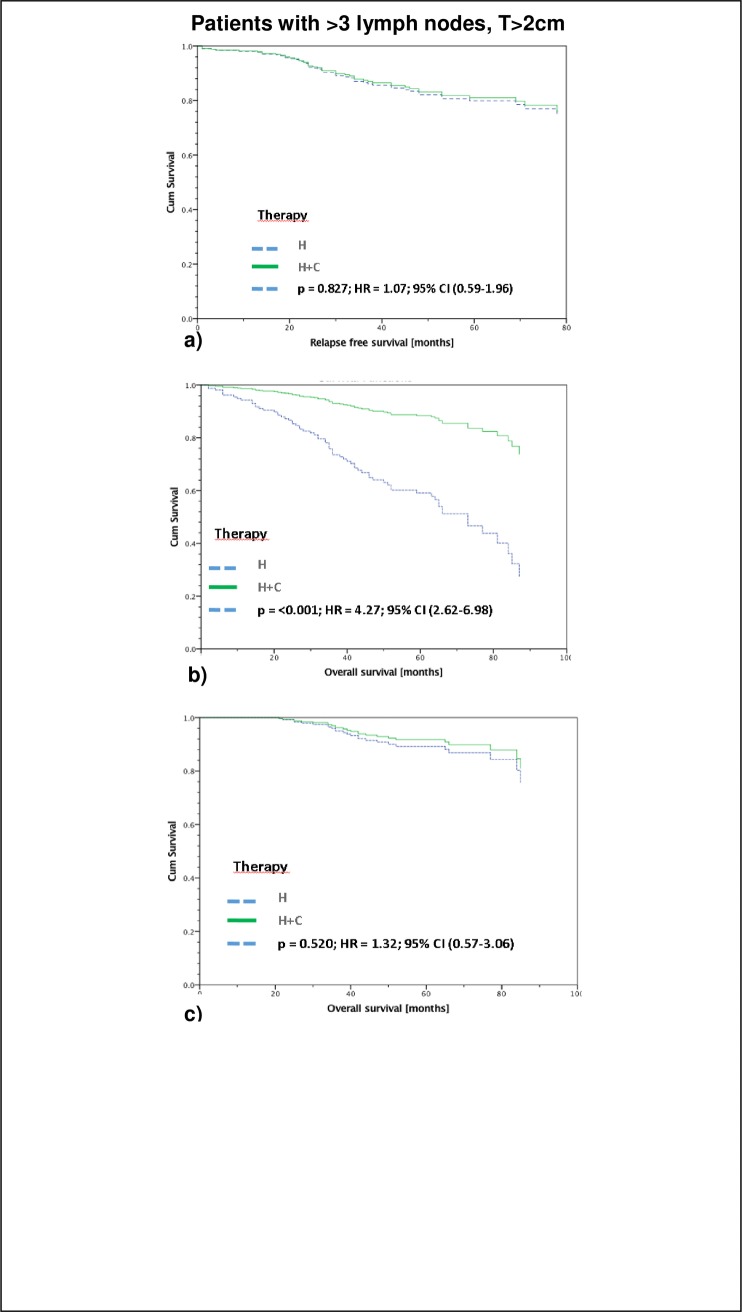
Patients > 3 lymph nodes and T> 2cm: Endocrine therapy vs. chemo- and endocrine therapy. Recurrence Free Survival (a), OAS (b), and tumorspecific OAS (c) of Luminal A patients with tumor size > 2cm and > 3 affected lymph nodes stratified by therapy and adjusted by age. There is no significant difference in RFS (a) (5-year RFS: H 80,0%; H+C 81,0%) and tumorspecific OAS (c) (5-year OAS: H 89,3,0%; H+C 91,7%) but a significant difference in OAS of all patients (b) (5-year tumor induced OS: H 59%; H+C 88%), n = 326.

Obviously, despite the worse prognosis of the 326 patients with larger tumors and more than 3 positive lymph nodes, adjuvant chemotherapy failed in order to improve survival of those patients. There was no significant difference in RFS (5-year RFS: endocrine therapy 80,0%; chemo- and endocrine therapy 81,0%) and tumorspecific OS (5-year OS: endocrine therapy 89,3,0%; chemo- and endocrine therapy 91,7%). However, regarding the OS including the non-tumorspecific survival, a significant improved survival has been observed in the group of patients, having received chemo- and endocrine therapy (59% vs. 88%; p>0,001) (Fig 4A–4C).

### Adjusted survival curves and log-rank test based on inverse probability weighting (IPW)

**Fig 5:**
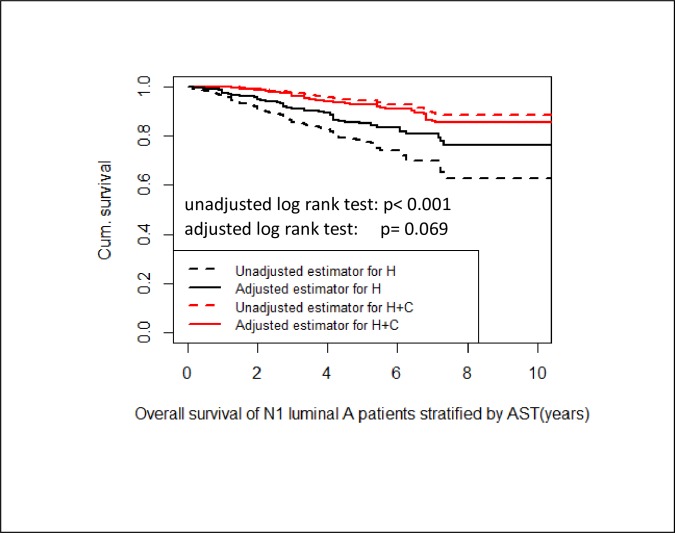
Adjusted survival. Unadjusted and adjusted Kaplan-Meier curves stratified by nodal positive luminal A patients with endocrine therapy and patients with endocrine and chemotherapy. The adjusted log rank test shows no significant difference in OS between both groups (p = 0.069).

In order to reduce imbalance in the empirical distribution of the pre-treatment confounders age, tumor size and number of affected lymph nodes we used propensity score methods The corresponding adjusted log rank test shows no significant difference in OS between luminal A N1 patients with endocrine therapy and patients with endocrine and chemotherapy (p = 0.069) (Fig 5).

## Discussion

In this retrospective study, we investigated the role of adjuvant systemic therapy in patients with primary nodal positive luminal A breast cancer. The main findings are: (1) Nodal positive luminal A breast cancer patients have in general a good to excellent prognosis (5-year RFS: 91% and tumorspecific 5-year OS 96.5%). (2) There was no significant difference in RFS stratified by patients with only endocrine therapy and with endocrine plus chemo-therapy. (3) For luminal A patients with 1–3 affected lymph nodes there was no significant difference in OS between patients with only endocrine therapy and with endocrine plus chemo-therapy, independent of tumor size. At first sight, the situation seems to be more difficult for patients with more than 3 affected lymph nodes (4). For luminal A patients with more than 3 affected lymph nodes we had a significant better OS for patients with endocrine plus chemo-therapy compared to patients with only endocrine therapy. (5) In contrast to the result in (4), we found no significant difference in tumorspecific OS between luminal A patients with more than 3 affected lymph nodes and only endocrine therapy and those with endocrine plus chemo-therapy. (6) After reducing imbalance in the empirical distribution of the pre-treatment confounders we could not find a significance in OS between the two therapeutic groups. (7) Patients with large tumors and more than three affected lymph nodes had a worse RFS and OS as compared to smaller tumors.

The difference between tumorspecific and non-tumorspecific OS stratified by AST can be explained by the fact that patients with only endocrine therapy were significantly older than patients with chemo- and endocrine therapy (median age 75y vs 57y). This imbalance is a bias.

For nodal positive luminal A patients tumor size and number of positive lymph nodes are important prognostic factors for the individual outcome. However, despite the worse survival of patients with larger tumors and highly nodal positive disease, adjuvant chemotherapy was not able to improve the outcome in terms of survival.

In these days, the selection of adjuvant systemic therapy in patients with primary breast cancer depends on genomic cancer subtypes as well as clinical parameters such as grading, tumor size or nodal status [1]. In the last decade, the use of adjuvant chemotherapy in general is gone back in favor of a more specific and precise application as a function of tumor biology. It is well accepted, that luminal B, HER-2 positive as well as triple negative or HER2 overexpressing breast cancer patients should receive (neo-) adjuvant chemotherapy. In addition, there is consensus, that nodal negative luminal A cancer patients only need adjuvant endocrine therapy. However, for the subgroup of (highly) nodal positive luminal A cancer, the benefit of adjuvant chemotherapy is an unanswered question.

Without any doubt, many important studies for the entirety of nodal positive patients have been published, supporting the use of adjuvant chemotherapy, even as dose-dense and/or dose-intense chemotherapy [16–24]. However, most of those studies did not discriminate the different molecular subtypes. For example, the ETC study included 25% of patients with HER2-positive cancer as well as hormone-receptor negative tumors [25]. Therefore, based on this data, it is hard to evaluate the impact of adjuvant chemotherapy of the subgroup of nodal positive luminal A cancer. This implicates that treating patients with nodal positive luminal A breast cancer will lead us in a therapeutic dilemma, on the one hand from a tumorbiological point of view, those patients do not benefit from a adjuvant chemotherapy, on the other hand with regards to the nodal status as important prognostic factor, patients with positive nodal status do need adjuvant chemotherapy. In this study we evaluated this question in order to dissolve this conflict.

First, it has been shown, that RFS in the entire study population did not differ significantly between the patients treated with adjuvant chemotherapy as compared to only endocrine treated patients. However, OS was significantly different. At first glance this result might be suggestive for a benefit of adjuvant therapy. However, since the mean age differs significantly between the group of endocrine treated vs. endocrine and chemotherapy treated patients, we assumed a bias. Presumably, the patients who received only endocrine had a worse OS due to fact that the median age is 75 years compared to 57 years for patients with additional chemotherapy, i.e. we have an imbalance in the empirical distribution of the pre-treatment confounders as result of the observational study. After reducing this imbalance by more advanced methods (propensity score methods), it turned out that there was no significant difference in OS between these two groups. This result is supported by the fact that we could not find any significant difference in tumor-specific overall survival. Despite the fact, that our analysis is based only on retrospective data, this findings support the hypothesis, that use of adjuvant chemotherapy in nodal positive luminal A patients should be discussed critically. In order to investigate the role of adjuvant chemotherapy in those patients properly, prospective data is needed.

This interpretation of our results is supported by others, who also detected no benefit of adjuvant chemotherapy in luminal A breast cancer patients [7, 26–30].

However, in contrast to the previously published data by Diessner et al. [26], we focused on nodal positive luminal A breast cancer and analysed the groups of 1–3 and more than 3 positive lymph nodes as well as the tumor size. Since it has been hypothesized, that in general larger tumors might be associated with a poorer outcome, small (T1) have been compared with larger tumors (T2) with regard to the subgroup of nodal positive patients with only 1–3 positive lymph nodes. Against all odds there was no significant difference in terms of RFS or OS, neither in the entire group of patients, nor in the chemotherapy treated patients. This observation indicates that at least in patients with nodal positive luminal A breast cancer with only 1–3 lymph nodes, tumor size seems not to be a relevant prognostic factor. This result is in line with others, who also distanced themselves from tumor size in favor of tumor biology in terms of evaluating the individual risk and prognosis of breast cancer patients [31, 32]. However, in our study population, patients with large tumors and more than three affected lymph nodes had a worse RFS and OS as compared to smaller tumors. This result has been observed in the entire group of patients (all therapies) as well as looking at the chemotherapy- treated patients. This surveillance raises the question, whether tumor size and nodal status in those patients act synergistically in terms of reduced survival or maybe if those larger luminal A tumors which initiate a highly positive nodal status represent a certain subgroup of luminal A cancers which is more aggressive. If the latter is the case, it can further be discussed if the worse prognosis and the highly positive nodal status are just associated or if the positive lymph nodes are a step on the way to metastasis and bad prognosis.

In either case, it is obvious that patients with large luminal A tumors with more than three positive lymph nodes have a reduced RFS and OAS as compared to early stage disease. Therefore the question if adjuvant chemotherapy improves survival rates in this group of patients has to be addressed. In our analysis we compared survival rates of patients with large tumors and more than three positive lymph nodes as a function of adjuvant chemotherapy. In our study population adjuvant chemotherapy in addition to endocrine therapy was not able to improve RFS and tumorspecific OS. Indeed, a significant difference in OS (non-tumorspecific) has been observed, but this effect seems to be a bias and is explainable due to the differences of median age in the chemotherapy vs. non-chemotherapy group. However, this observation has to be interpreted carefully, since the database did not differ between conventional and dose-dense/dose-intensive chemotherapy, which might improve survival also in luminal A patients. Therefore, the question of the meaning of dose-dense/dose-intensive chemotherapy in nodal positive luminal A breast cancer remains uncleared.

In summary, our data provide evidence that nodal positive patients with luminal A breast cancer have, if any, a limited benefit of adjuvant chemotherapy. Tumor size and nodal status seem to be of prognostic value in terms of survival, however at least in our study population both tumor size as well as nodal status was not predictive for a benefit of adjuvant chemotherapy which might be change clinical practice in future.
